# Climate and air pollution impacts on habitat suitability of Austrian forest ecosystems

**DOI:** 10.1371/journal.pone.0184194

**Published:** 2017-09-12

**Authors:** Thomas Dirnböck, Ika Djukic, Barbara Kitzler, Johannes Kobler, Janet P. Mol-Dijkstra, Max Posch, Gert Jan Reinds, Angela Schlutow, Franz Starlinger, Wieger G. W. Wamelink

**Affiliations:** 1 Department for Ecosystem Research and Environmental Information Management, Environment Agency Austria, Spittelauer Lände 5, Vienna, Austria; 2 Austrian Research Centre for Forests—BFW, Vienna, Austria; 3 Alterra, Wageningen UR, Wageningen, the Netherlands; 4 Coordination Centre for Effects (CCE), RIVM, Bilthoven, the Netherlands; 5 OEKO-DATA—Ecosystem Analysis and Environmental Data Management, Strausberg, Germany; Chinese Academy of Forestry, CHINA

## Abstract

Climate change and excess deposition of airborne nitrogen (N) are among the main stressors to floristic biodiversity. One particular concern is the deterioration of valuable habitats such as those protected under the European Habitat Directive. In future, climate-driven shifts (and losses) in the species potential distribution, but also N driven nutrient enrichment may threaten these habitats. We applied a dynamic geochemical soil model (VSD+) together with a novel niche-based plant response model (PROPS) to 5 forest habitat types (18 forest sites) protected under the EU Directive in Austria. We assessed how future climate change and N deposition might affect habitat suitability, defined as the capacity of a site to host its typical plant species. Our evaluation indicates that climate change will be the main driver of a decrease in habitat suitability in the future in Austria. The expected climate change will increase the occurrence of thermophilic plant species while decreasing cold-tolerant species. In addition to these direct impacts, climate change scenarios caused an increase of the occurrence probability of oligotrophic species due to a higher N immobilisation in woody biomass leading to soil N depletion. As a consequence, climate change did offset eutrophication from N deposition, even when no further reduction in N emissions was assumed. Our results show that climate change may have positive side-effects in forest habitats when multiple drivers of change are considered.

## Introduction

Climate change combined with excess deposition of nitrogen (N) are among the main stressors of biodiversity, at least in Europe, North America and parts of Asia [1]. Climate warming has caused phenological, physiological and genetic adaptations and changes of spatial distribution patterns of plant and animal species [2, 3]. Nutrient enrichment in response to N deposition has also caused changes in the structure of communities and declines in biodiversity [4]. According to large-scale modelling, major parts of the European Natura 2000 habitats, which are at the core of the European Habitat Directive [5], are under threat [6]. If both greenhouse gas emissions, causing climatic warming, and N emissions from fossil fuel burning and agriculture, leading to the deposition of reactive N, are not mitigated more effectively in the future, even harsher effects are to be expected [7–9]. Some success in reducing N emissions has already been achieved in North America and Europe. As a result, total N in wet deposition in Europe decreased on average by 2.7% between 2001–2002 and 2005–2007 [10]. However, no climate change mitigation measures are in place at a scale necessary to avoid further damage [11] nor will N emission reductions be able to fully release sensitive habitats from chronic N stress [6]. Moreover, N deposition in South America, Africa and most prominently in Asia are expected to raise further [10]. That is why studying the magnitude of these threats to protected habitats is important.

To date, assessments of potential future risks to biodiversity have largely neglected interactions between climate change, N deposition, and biodiversity [12]. Since most plant species have a distinct climate niche, climate warming and changes in the precipitation regime alter the distribution pattern of their suitable habitats and eventually lead to the loss of species [13–15]. The main effect due to increased N deposition in terrestrial ecosystems is enhanced growth of some species, effectively using the additional N [4, 16]. In forests, increasing availability of soil N for plants might lead to more homogenous forest floor plant communities and hence to biodiversity loss [17] which in turn might have consequences for the food web structure and ecosystem functioning [18]. Beside deposition of inorganic N compounds, plant N availability is controlled by the effects of temperature and moisture on litter decomposition rates and mineralization of organic N soils [19]. Whereas N deposition leads to an increase in N availability [20–24], expected climate change may have different effects. Warming will lead to enhanced N mineralization and nitrification and eventually to higher N uptake in response to higher tree growth [25, 26]. McDonnell et al. [27] showed in a modelling study that tree growth stimulation by climate change can considerably offset negative effects of N deposition on biodiversity. Wamelink et al. [28] showed that a higher N deposition may lead to devastating effect on biodiversity, especially in heathland and semi-natural grasslands.

Although we focus on eutrophication effects of N deposition and climate change induced impacts, soil acidification (through N and sulphur deposition) is an important additional driver. Sulphur (S) deposition (and to some extent N) has led to large-scale soil acidification during the second half of the 20^th^ century, but significantly decreased in Europe since the late 1980s [10] followed by increasing soil pH values [29, 30] and a decrease in the prevalence of plant species adapted to acid soils [31–33]. Climate warming can potentially accelerate soil recovery from acidification, because base cation input to the soil increases with an increase in weathering and litter decomposition [34, 35].

Coupled soil-plant models capable of quantifying potential interacting effects of climate change and N deposition on biodiversity are just emerging [36] and are being increasingly applied [27, 35, 37–39]. Here we used the dynamic biochemical soil model VSD+ [40] together with two plant response models, PROPS [41] and BERN (for evaluation only) [39] with data from 18 long-term forest ecosystem observation sites distributed across Austria. Both plant models use empirical environmental response functions to predict species occurrence based on its realized niche [42]. We focused on habitat suitability, i.e. the capacity of a site (habitat) to host its characteristic plant species relying on the concept of potential natural vegetation [43]. We do so because the European Habitat Directive [5] adheres to the concept of “favourable conservation status” which can be measured by habitat suitability [44]. For future predictions three different N and S deposition and four different climate scenarios were used. Specifically, we hypothesized 1) that climate change will weaken the suitability of forest habitats to host their characteristic plant species because major changes in temperature and precipitation will push these species out of their optimum range, 2) that N deposition will also lower the habitat suitability due to soil N enrichment and a concomitant increase in the occurrence probability of nitrophilic plant species on the expense of oligotrophic species, and 3) that when taking interactions into account, eutrophication in forest floor species composition due to N deposition effects will be mitigated by climate driven increased N immobilisation in wood biomass.

## Methods

The field sites are all part of the Austrian federal long-term monitoring program of air pollution effects of the Austrian Ministry for Agriculture, Forestry, Environment and Water Management. Owners of the land gave permission to conduct monitoring. Our study did not involve field studies on endangered species other than observation no involvement of human participants, specimens or tissue samples, or vertebrate animals, embryos or tissues.

### Site characteristics

We used 18 forest sites which are part of ICP Forests and ICP Integrated Monitoring program within the framework of effects monitoring of the UNECE Convention on Long-range Transboundary Air Pollution [45] (Table 1). These sites were located to represent the variation in the more widespread forest ecosystems in Austria and hold long-term measurements of climate parameters, stand properties and soil condition [46–48].

**Table 1:** Study sites, their code and name according to the European Habitat Directive (Directive 92/43/EEC), and their potential natural vegetation types. Characteristic plant species are listed in S1 Table.

EU Habitat type	Site code	Altitude (m)	Lat	Lon	Potential Natural Vegetation
9110: Luzulo-Fagetum beech forests	IF_AT05	720	46.72	13.68	Luzulo-Abieto-Fagetum (typ. Subass.) HARTM. et JAHN 1967
	IF_AT08	630	48.93	15.19	Calamagrostio villosae-Fagetum syvatici MIKUSKA 1972
	IF_AT10	960	48.1	12.87	Luzulo-Abieto-Fagetum (typ. Subass.) HARTM. et JAHN 1967
	IF_AT13	670	46.63	15.52	Luzulo-Abieto-Fagetum sylvatici (Dryopteris-Subass.) HARTM. et JAHN 1967 and Asperulo-Abieti-Fagetum sylvatici (Dryopteris-Subass.) TH. MÜLLER 1966
9130: Asperulo-Fagetum beech forests	IF_AT03	930	46.74	14.50	Asperulo-Fagetum SOUGNEZ et THILL 1959
	IF_AT04	1190	46.77	13.17	Asperulo odoratae-Fagetum SOUGNEZ & THILL 1959
	IF_AT09	510	48.12	16.05	Hordelymo-Fagetum sylvatici TX. 1937 (Dryopteris-Subass.)
	IF_AT11	860	47.88	13.35	Asperulo-Abieti-Fagetum sylvatici (Dryopteris-Subass.) TH. MÜLLER 1966 and Luzulo-Abieto-Fagetum (typ. Subass.) HARTM. et JAHN 1967
	IF_AT15	715	47.63	15.66	Helleboro nigri-Fagetum sylvatici ZUKRIGL 1973
	IM_AT01	900	47.84	14.44	Cardamino trifoliae-Fagetum sensu WILLNER 2002
	IM_AT02	880	47.84	14.44	Adenostylo glabrae-Fagetum sensu WILLNER 2002
9150: Medio-European limestone beech forests of the Cephalanthero-Fagion	IF_AT07	500	47.65	16.13	Cyclamini (purpurascentis)-Fagetum sylvatici SOÓ 1962
91G0: Pannonic woods with Quercus petraea and Carpinus betulus	IF_AT01	390	47.77	16.32	Carici pilosae-Carpinetum NEUH. & NEUH.-NOV.1964
	IF_AT02	290	47.49	16.56	Sorbo torminalis-Quercetum (petraea) SVOBODA ex BLAZKOVA 1962 incl. Festuco heterophyllae-Quercetum Neuh. & Neuh.-Nov. 1964
9410: Acidophilous Picea forests of the montane to alpine levels (Vaccinio-Piceetea)	IF_AT16	1540	47.06	14.11	Homogyno alpinae-Piceetum (Rhytidiadelphus loreus-Subass.) ZUKRIGL 1973
	IF_AT18	1020	47.39	10.91	Calamagrostio variae-Piceetum SCHWEINGRUBER 1972
	IF_AT12	920	47.49	13.42	Bazzanio-Piceetum (SCHMIDT et GAISBERG 1936) BR.-BL. et SISSINGH in BR.-BL. et al. 1939
	IF_AT14	960	47.37	15.17	Galio rotundifolii-Abietetum WRABER 1959

The sites comprise 5 forest types protected under the European Habitat Directive in Austria. These are:

- *Luzulo-Fagetum* beech forests (EU code 9110) with *Fagus sylvatica L*. as the dominant tree species, found at submontane to montane altitudes on acid soils in the Austrian Alps, its foothills, and the Bohemian Massive;
- *Asperulo-Fagetum* beech forests (EU code 9130) with *Fagus sylvatica* as the dominant tree species found on neutral or near-neutral soils with mull humus;
- Medio-European limestone beech forests of the *Cephalanthero-Fagion* (EU code 9150) with *Fagus sylvatica* as the dominant tree species found on dry south slopes at submontane to lower montane altitudes with base-rich soils on carbonate bedrock;
- Pannonic woods with *Quercus petraea (Matt*.*) Liebl*. and *Carpinus betulus L*. *(EU code 91G0)*, which are mixed deciduous forests found at colline to submontane altitudes of eastern Austria with dry continental climate;
- Acidophilous *Picea* forests of the montane to alpine levels (*Vaccinio-Piceetea*) (EU code 9410) with *Picea abies (L*.*) H*.*Karst*. as the dominant tree species found at montane and subalpine altitudes of the Austrian Alps.

### Soil-vegetation model

**Fig 1:**
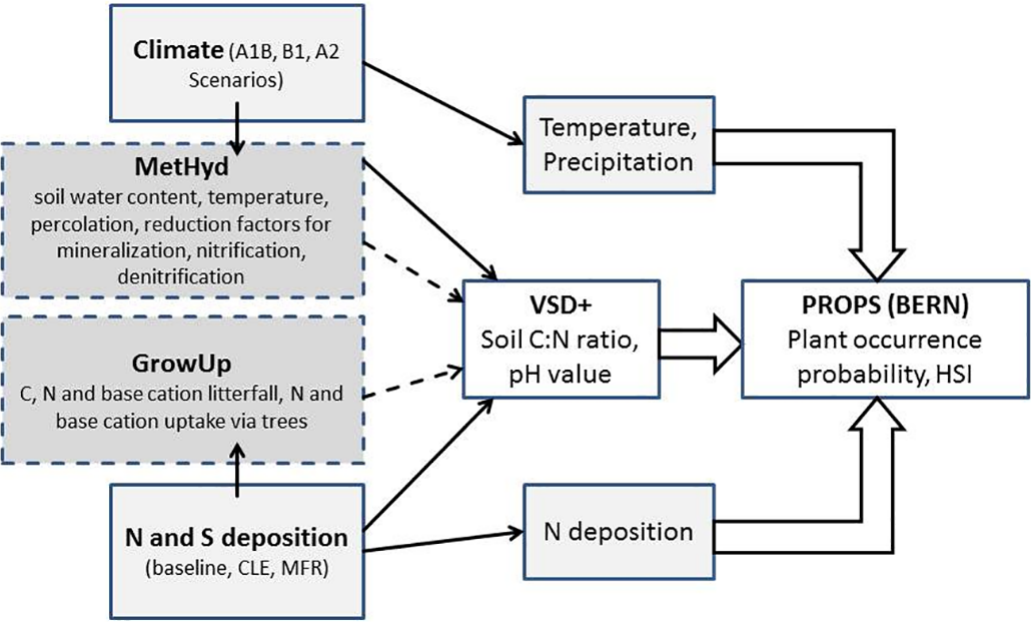
Model chain. Model chain with VSD+ as the dynamic soil chemistry model and PROPS model for predicting the probability of occurrence for all characteristic species (BERN model for validation) and its respective Habitat Suitability Index (HSI). Climate driven inputs to VSD+ came from the hydrological model MetHyd, litter element input and plant uptake were calculated with GrowUp. Climate, N and S deposition scenario data was input to the model chain.

We use the dynamic geochemical soil model VSD+ (version 5.4, [40]) together with a newly developed plant response models PROPS [41]. For validation purpose, we also applied the plant model BERN [39] (Fig 1). The VSD+ model includes cation exchange (Gaines-Thomas or Gapon) and organic C and N dynamics according to the RothC-Model (version 26.3, [49]). VSD+ is driven by time series of N and S deposition as well as temperature and hydrology to predict soil solution chemistry and C and N pools. In its current version, the PROPS model is a database holding statistical niche functions for 4053 plant species occurring in Europe that were derived from a huge set of vegetation relevés together with associated soil data [41]. The outputs of PROPS are probabilities of species occurrences as a function of precipitation, temperature, N deposition, soil C:N ratio and soil pH. As the final habitat response indicator, we applied the Habitat Suitability Index (HSI) that describes the degree of suitability of site conditions for a set of typical species to co-occur. The HSI is defined as the arithmetic mean of the normalised probabilities of occurrence of these species [6]. In our study, we adopted the common approach taken in the EU [50] to use phytosociological plant community descriptions to define distinctive plant species for each of the 18 forest ecosystems (see Table 1 and S1 Table). These distinctive species are the characteristic species and all constant attendant species that can be found with a similar abundance in more than 70% of all vegetation relevés representing the plant community.

### Model setup

We had climate measurements with a daily resolution at the sites (6 sites) or from the closest meteorological station representative for the site (12 sites). These time series comprised the last 20–27 years. By using the weather generator ClimGen [51] and this measured data (precipitation, maximum temperature, minimum temperature, air humidity, global solar radiation), we derived the baseline climate time series for 1950–2100. Then, scenario data were synthesized by means of anomalies gathered from the A1B, A2 and B1 scenarios [52]. Newer, downscaled climate scenarios were not available at the start of the project. Parameter-specific monthly climate change anomalies for the study site were derived from the respective grid cell of the regional climate model COSMO-CLM [53]. The A1B, A2, and B1 scenarios were based on the global circulation model ECHAM5 and the A1B scenario was also available from the HadCM3 model [52] (Table 2).

**Table 2:** Climate change, N and S deposition scenarios. Mean change and standard error in temperature (T) and precipitation (P) for three time slices (2030: 2025–35; 2050: 2045–55; 2100: 2090–2100) as compared to 2010 (2005–15). N and S deposition for 2020 and after 2030 with three scenarios given as B10: N and S deposition in the year 2010 with no further reduction; CLE: N and S deposition under revised Gothenburg Protocol emissions; MFR: N and S deposition under the technically maximum feasible emission reductions.

GCM	Scenario	2030		2050		2090	
		T (°C)	P (mm)	T (°C)	P (mm)	T (°C)	P (mm)
ECHAM5	A1B	0.2 ±0.05	52 ±21	1.8 ±0.06	-21 ±16	3.4 ±0.1	-24 ±33
	A2	0 ±0.05	-56 ±21	1.1 ±0.05	-38 ±14	4 ±0.09	-140 ±27
	B1	0.3 ±0.06	41 ±18	0.5 ±0.06	100 ±17	2.1 ±0.08	-109 ±22
HadCM3	A1B	1.2 ±0.06	-51 ±18	1.7 ±0.07	64 ±17	3.9 ±0.09	-123 ±24
		2030–2090					
		N (kg ha^-1^ yr^-1^)			S (kg ha^-1^ yr^-1^)		
	B10	-0.2 ±0.14			-0.5 ±0.06		
	CLE	-2 ±0.23			-1.4 ±0.12		
	MFR	-4 ±0.6			-1.5 ±0.14		

We calculated total deposition of N and S for each site as the sum of throughfall and canopy exchange. The method is based on a canopy exchange model according to [54] with sodium as the tracer ion. In order to get long-term deposition of N and S we scaled large-scale modelled data to the measurements. We used reconstructed deposition from 1880 to 2000 [55] and deposition values for 2005, 2010, 2020 and 2030 from the latest EMEP model version [56] using the current legislation (CLE) scenario with revised Gothenburg Protocol emissions and the technically maximum feasible emission reduction scenario (MFR). A third deposition scenario was kept constant after 2010 (B10) (Table 2).

Soil data were taken from soil inventories in 2005 (IM_AT01, IM_AT02) or 2008 (all other sites). Mineral soils were sampled at depths of 0–5, 5–10, 10–20, 20-(35)40, and (35)40-(50)80 cm from a number (n≥3) of soil pits per plot. The organic layer was sampled and analysed separately. Specimen preparation and analysis followed the methods described in the ICP Forests monitoring program (http://icp-forests.net/page/icp-forests-manual). The calculation of input parameters for VSD+ followed the recommendations given in [40] and are described in more detail in S2 Table (input files in S1 File). Soil solution element concentrations were available for the 6 evaluation sites and were aggregated to mean annual values for the years 1998 to 2012.

Uptake of C, N and base cations as well as above- and belowground litterfall was calculated using GrowUp, version 1.3.2 [40]. Since both climate change and nitrogen deposition are supposed to change tree growth and thereby the uptake of N by trees and input via litterfall, a scaling of these input variables was done comparable to [57]. N and base cation uptake as well as C and N in litterfall were scaled according to a reference situation, i.e. mean values between 1970 and 1990 where the core of the forest yield tables [58] was obtained (see details in S2 File).

For 6 sites detailed data on forest floor vegetation was available for several years between 1990 and 2010 [31]. Forest vegetation at the study sites was recorded on permanent plots using sub-sampling units which were selected randomly or were distributed in a regular grid. Permanent plot size was 0.5 ha and sub-sampling unit size was 4 m^2^. For the ICP Integrated Monitoring plots IM_AT01 and IM_AT02 8 subsamples per plot were recorded, for ICP Forests sites 10 subsamples were available.

### Model calibration and evaluation

The soil model VSD+ was calibrated using measured C and N pools, base saturation and soil solution chemistry (pH for all sites; NO_3_^-^ and SO_4_^2-^ for 6 sites) using Markov Chain Monte Carlo method [59]. Linear regression, mean error and RMSE between measured and modelled values were calculated for soil C pools, C:N ratios, and soil solution pH, SO_4_^2-^ and NO_3_^-^. We calculated observed HSI by using the frequency of characteristic species in vegetation records of the sites. Additionally, we compared results from PROPS with the HSI calculated with the BERN model. BERN differs from PROPS as to the underlying relevé data and regarding the statistics used in deriving niche functions [36, 39]. Linear regressions between the two models were calculated.

### Statistical analyses

In total, three N and S deposition scenarios (B10, CLE, MFR) and 5 climate scenarios (including baseline) were modelled for 18 sites resulting in 270 datasets.

First, we applied a two-way ANOVA with F-test statistics to test whether an overall trend exists over time in temperature (T), precipitation (P), N and S deposition, soil C:N ratio, soil solution pH and HSI and whether this trend is different between the climate scenarios and the deposition scenarios. To account for repeated measures over time, the scenarios were nested into four time slices (2010: average of 2005–15; 2030: 2025–35; 2050: 2045–55; 2100: 2090–2100) and interactions between climate and deposition scenarios were taken into account. Second, we used the same ANOVA design for each site. The ANOVA coefficients were used to determine effect sizes of the climate and deposition scenarios on soil C:N ratio, soil solution pH and HSI. Differences in effect sizes by 2100 between EU protection habitat types were tested using one-way ANOVA and Tukey’s HSD multi-comparison test. All statistical analyses were carried out with the package R, version 3.2.3 [60].

## Results

### Model calibration and evaluation

**Fig 2:**
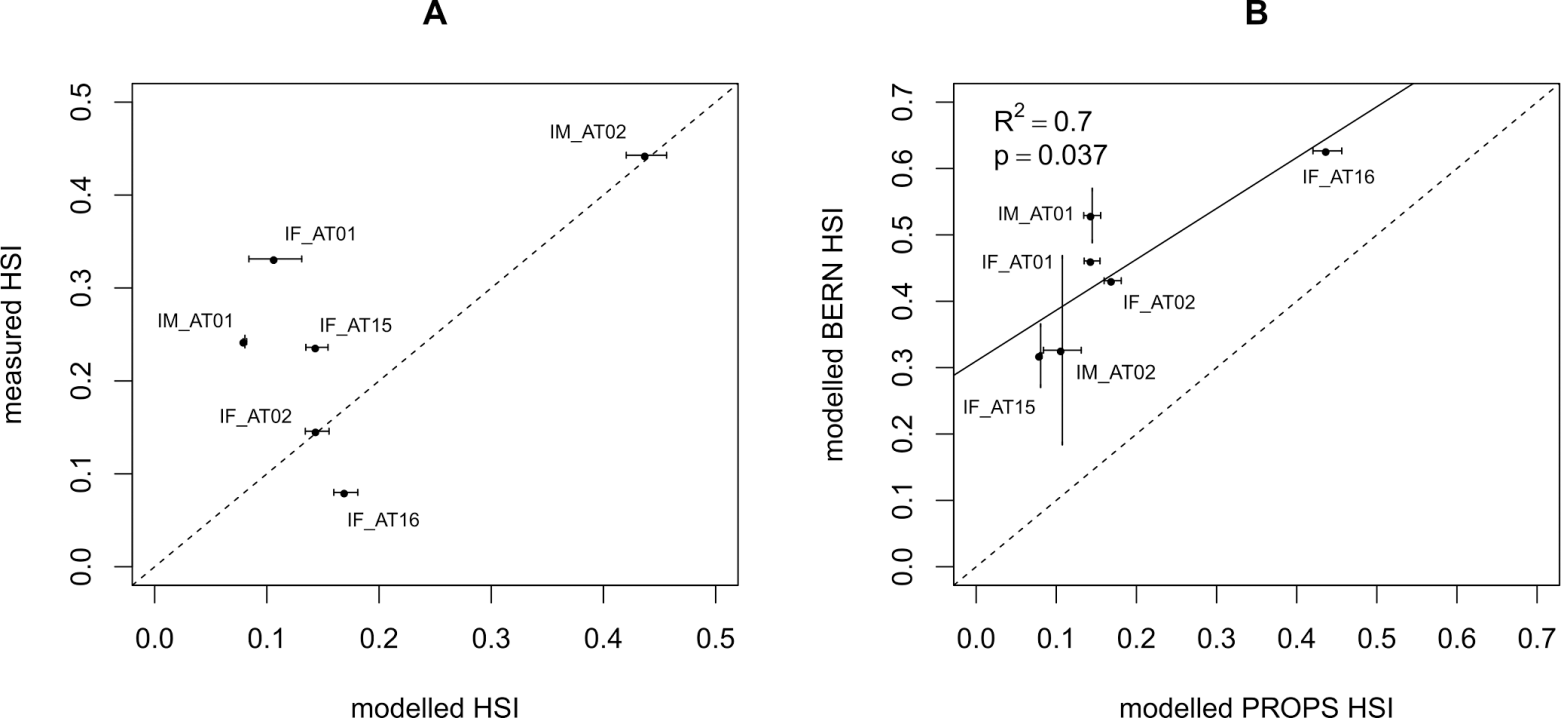
Modelled versus observed Habitat Suitability Index (HSI). Comparison of the modelled Habitat Suitability Index (mean and standard deviation) using PROPS with A) observed data and B) with modelled HSI from BERN (using the same soil and climate data as PROPS). Six sites with vegetation records between 1996 and 2007 were used. The 1:1 line is dashed, the regression line is solid.

Using linear regression, R^2^ between modelled and measured HSI was 0.36 but not significant (p = 0.201) (Fig 2). Mean error was 0.05 and RMSE was 0.12. HSI values as derived from VSD+ and PROPS were comparable with those derived from VSD+ and BERN (R^2^ = 0.70, p = 0.037) but they differed in magnitude, with PROPS giving lower values (mean error was 0.22 and RMSE was 0.26) (Fig 2).

Modelled and measured forest growth achieved an R^2^ of 0.63 (p<0.001), mean error was 527 kg m^-2^ and RMSE was 5519. VSD+ could be calibrated to the observed C pool and C:N-ratios resulting into R^2^ of modelled versus measured values of 0.86 and 0.99, respectively (p-values < 0.001). The mean error for the C pool was 1.05 kg m^-2^, RMSE was 2.19. The mean error for the C:N ratio was 0.13, RMSE was 0.68. As to soil solution composition, only pH values were significantly related with observations (R^2^ = 0.57, p<0.001; mean error = 0.41, RMSE = 1.32). SO_4_^2-^ and NO_3_^-^ had mean errors of 0.15 and 0.05 eq m^-^^3^ and RMSE of 0.21 and 0.07 (S3 File).

### Future trends in climate and deposition

Future T and P changed significantly over time in the applied climate scenarios (S4 File). Whereas T increased by 2.7°C on average between 2010 and 2100, P increased slightly towards 2050 and decreased by 76 mm in the year 2100 in comparison to 2010 (Table 2). Only T trends were significantly different between the climate scenarios. The ECHAM5-A1B, A2 and HADCM3-A1B scenario showed the highest T increases (3.4–4°C by 2100 on average), whereas the ECHAM5-B1 scenario showed the lowest increase (2.1°C). Only in ECHAM5-A2 P decreased continuously. ECHAM5-A1B and HADCM3-A1B were relatively similar regarding their T trajectories (the latter showed a faster increase) but differed as to P. Neither increase in T until 2100 nor change in P were significantly different between habitat types (ANOVA p = 0.979 and 0.364).

In both, the current legislation scenario (CLE) and the maximum feasible reduction scenario (MFR), N and S deposition decrease significantly over time and significantly differed from each other (S4 File). Averaged over all sites, N deposition decreased by 2 kg N.ha^-1^.yr^-1^ (CLE) and 4 kg N.ha^-1^.yr^-1^ (MFR) compared to the deposition in 2010 (Table 2). Differences in the decrease in N deposition until 2100 between habitat types due to regional differences in deposition trajectories were marginally significant (ANOVA p = 0.099) due to habitat type 9410 which experienced stronger decrease than 9130 (Tukey’s HSD p = 0.053).

### Effects of climate, N and S deposition

**Fig 3:**
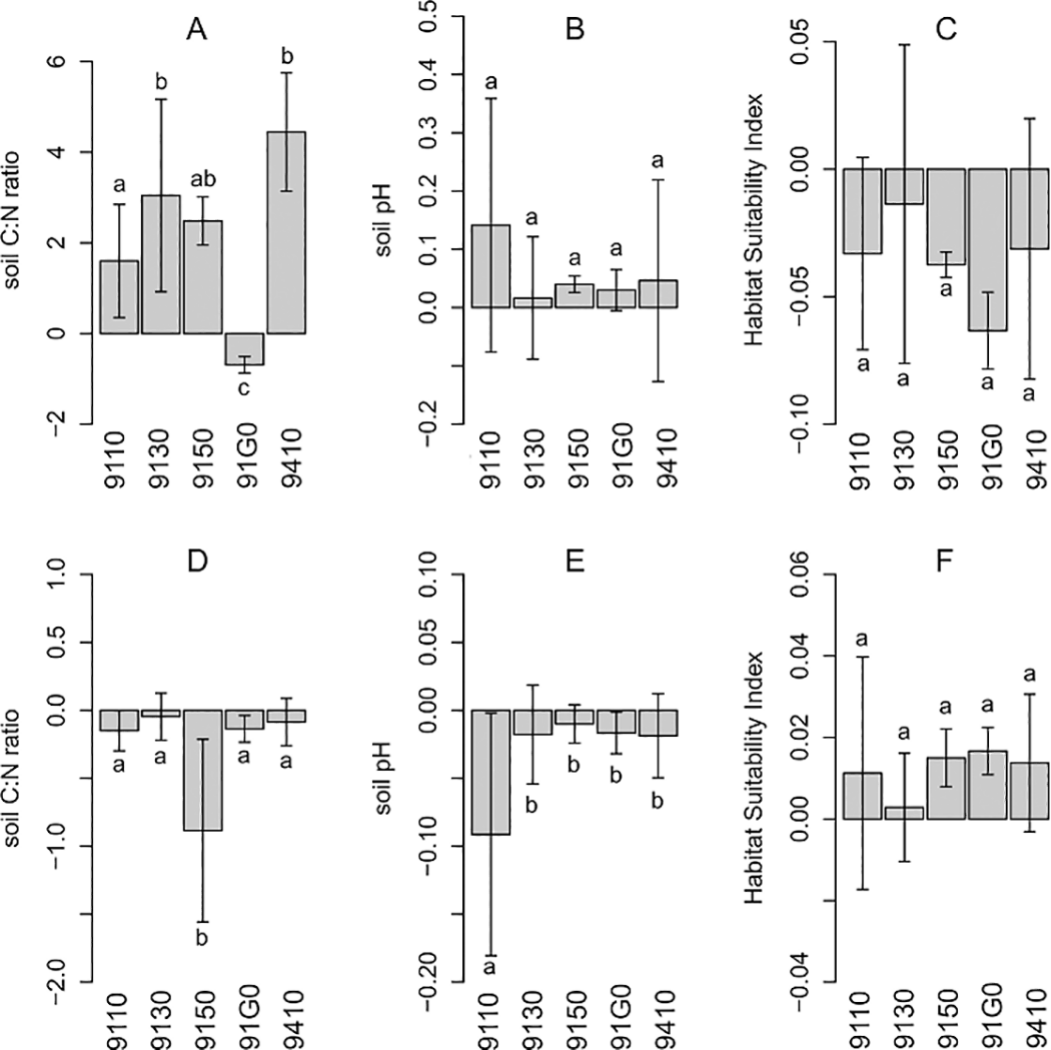
Effects of climate change and N deposition scenarios. Effects of climate change (A-C) and N and S deposition (D-F) on soil C:N ratio, soil pH, and the Habitat Suitability Index (mean and standard deviation of effects in the year 2100 as derived from 5 different climate change scenarios). Means with different letters are significant different (Tukey’s HSD p < 0.05). Effects are given in the form of ANOVA coefficients describing the difference between the mean values of all baseline climate model runs and the respective climate change scenario by the year 2100 and the difference between the mean values of all MFR deposition scenarios and the respective CLE and B10 deposition scenario by the year 2100 at each site. Positive coefficients represent increasing, negative coefficients decreasing effects. Note that MFR scenarios have the lowest N deposition. 9110: Luzulo-Fagetum beech forests, 9150: Medio-European limestone beech forests of the Cephalanthero-Fagion, 9130: Asperulo-Fagetum beech forests, 91G0: Pannonic woods with Quercus petraea and Carpinus betulus, 9410: Acidophilous Picea forests of the montane to alpine levels (Vaccinio-Piceetea).

The average increase of the soil C:N ratio units until 2100 over all scenarios was 3.4 (p<0.001) (S4 File). When compared to the baseline climate, climate scenarios had an increasing effect on soil C:N ratios in 82% of the sites and years (2030, 2050 and 2100). HADCM3-A1B had the strongest effect on C:N ratios (median: 0.72; 90^th^ percentile: 4.29). Climate effects on C:N ratios by the year 2100 were different between habitat types (ANOVA p<0.001, Fig 3). B10 and CLE deposition scenarios, when compared to the lowest emission scenario (MFR), had decreasing effects on C:N ratios in 29% of all scenario runs and increasing effects in 69% (S3 Table). The effects of N deposition were an order of magnitude lower than the climate effects (B10: median = 0.03 and 90^th^ percentile = 0.15). Deposition effects on C:N ratios by the year 2100 were mostly negative and differed between habitat types (ANOVA p<0.001, Fig 3).

The average soil solution pH over all scenarios increased slightly between 2010 and 2100, but not significantly (p = 0.209) (S4 File). When compared to the baseline climate, climate scenarios had an increasing effect on soil solution pH in 50% of the sites and years (2030, 2050 and 2100) whereas 22% remained unaffected and in 28% we found a decreasing effect (S3 Table). Climate effects on soil pH by the year 2100 were indifferent between habitat types (ANOVA p = 0.136, Fig 3). Higher future N deposition had decreasing effects on soil pH in 57% of all model runs and no effect in 42%. Deposition effects on soil pH by the year 2100 were different between habitat types (ANOVA p<0.028, Fig 3). Overall, the effects of different climate and deposition scenarios on soil solution pH were rather small (10% percentile -0.11–0.02; 90% percentile: 0–0.18) (S3 Table).

The average HSI over all scenarios decreased significantly from 2010 to 2100 (from 0.2 to 0.14, p<0.001) (S4 File). When compared to the baseline climate, climate scenarios had a negative effect on HSI in 43% of the sites and years (2030, 2050 and 2100), 21% had a positive effect and 36% had no effect (S3 Table). Both A1B scenarios had the highest proportion of negative effects, ECHAM5-B1 the lowest. Positive and negative effects increased over the years so that only in 11–22% of the climate scenario runs in 2100 no effects were found. With higher deposition in B10 and CLE deposition scenario as compared to MFR, HSI was negatively affected in only 15%, positively affected in 45% and 40% of the model runs did not cause an effect. The effects of different climate and deposition scenarios on HSI were rather small (10% percentile of all model runs: -0.06 to -0.01; 90% percentile: 0.01–0.04) (S3 Table).

Climate effects on HSI by the year 2100 were indifferent between habitat types (ANOVA p = 0.255). However, for sites belonging to the EU habitat type 91G0, HSI exhibited the strongest negative climate effects (Fig 3) resulting from a disproportional number of plant species with decreasing occurrence probability, most strongly *Stellaria holostea L*. and *Carpinus betulus* (S5 File). Also sites belonging to EU habitat types 9110, 9150, and 9410 HSI experienced negative climate change effects on average. The plant species experiencing the strongest negative effects were (in descending order) *Linnaea borealis L*. and *Lonicera caerulea L*. (EU habitat type 9410), *Hieracium murorum L*. and *Luzula luzuloides (*Lam.*)* Dandy *&* Wilmott. (EU habitat type 9110), and *Mercurialis perennis L*. (EU habitat type 9150). In contrast to these habitats, mean effect on HSI in the EU habitat 9130 was small, and in some cases even positive (ECHAM5-B2 and HADCM3-A1B) (Fig 3). There, a small number of plant species showed strongly increasing occurrence probability, particularly in the habitat type 91G0 (*Rosa canina L*., *Hedera helix L*., *Quercus petrea*). The strongest negative effects were found for *Senecio ovatus (G*.*Gaertn*. *et al*.*) Willd*. and *Sorbus aucuparia L*., the strongest positive effects for *Huperzia selago (L*.*) Bernh*. and *Vaccinium myrtillus L*. (S5 File).

As mentioned above, HSI showed a positive response to higher N and S deposition in the B10 and CLE scenario as compared to the MFR scenario. Deposition effects on HSI by the year 2100 were indifferent between habitat types (ANOVA p = 0.742, Fig 3). The differences in the occurrence probabilities of plant species between the baseline climate and climate scenarios was much higher (between -60 and +30%) than the differences between deposition scenarios. MFR versus B10 scenarios resulted into differences in the range of -4 to +16%. The species with the strongest increases per habitat type were *Hieracium murorum* and *Vaccinium myrtillus* (EU habitat type 9110), *Carpinus betulus* and *Corylus avellana L*. (EU habitat type 91G0), *Milium effusum L*. and *Senecio ovatus* (EU habitat type 9130), *Carex sylvatica Huds*. and *Athyrium filix-femina (L*.*) Roth* (EU habitat type 9410), and *Fagus sylvatica* and *Mercurialis perennis* (EU habitat type 9150) (S5 File).

## Discussion

### Climate change effects on soil chemistry and HSI

According to our climate change scenarios, T increased and P decreased towards 2100 and these changes did not differ between forest habitat types albeit they differed between scenarios. When singled out, i.e. calculating differences between the baseline and climate change scenarios, climate strongly increased the soil C:N ratio. This increase in soil N deficiency was mostly triggered by N immobilisation in woody biomass as derived from empirical relationships between climate and forest growth [57]. Interactions of temperature and precipitation were taken into account insofar as to limit tree growth, particularly in relatively dry areas in eastern Austria which are likely becoming even more water deficient under expected climate change. As a consequence, the C:N ratio of oak forest (habitat 91G0) soils in these areas were negatively affected by climate change.

We expected that these climate changes should directly increase the occurrence probability of the most thermophilic plant species among the distinctive species of a habitat while the most cold-tolerant species should decrease. Indeed, the species experiencing the strongest negative climate effect were cold-tolerant species (e.g. *Linnaea borealis*, *Lonicera caerulea*) and many of those increasing were thermophilic (*Rosa canina*, *Hedera helix*). Additionally, and except for the dry forest habitat 91G0, oligotrophic species should increase while species preferring sites with higher nutrient availability should decrease. Likewise, oligotrophic species increased (e.g. *Vaccinium myrtillus*) but not so in the dry habitat 91G0. There, the thermophilic species of nutrient rich sites, *Stellaria holostea L*., increased. This is in accordance with decreasing soil C:N ratios indicating higher nutrient availability. Species such as *Hieracium murorum* and *Luzula luzuloides* decreased in acidophytic beech dominated forests (habitat 9110). These are typical species of these forests but future N availability may decline to an extent which lowers their abundance. These changes in species occurrence probabilities decreased the HSI in all habitats with no significant differences in the magnitude between them because direct climate effects on the habitat suitability predominated. To our knowledge, no comparable study exists so far. However, impacts studies have shown the decisive role of climate for the future of tree species in Austria [61] and neighbouring countries [62, 63].

### N and S deposition effects on soil chemistry and HSI

Contrary to our expectations, the response of the HSI to higher N and S deposition in the B10 and CLE scenario as compared to the MFR scenario was positive. We found a slightly increasing overall trend in soil solution pH, corroborating observations from the Austrian forest soil monitoring [64] and other long-term observations across Europe [29, 30, 65, 66]. The small change in soil pH was no surprise since some of the sites are well buffered, S deposition declined most strongly in the 1980s and is on a rather low level since several years [10], and N deposition continued to acidify soils. When singling out deposition effect on soil pH from this long-term trend, beech dominated forests on acidic soils (9110), were more affected than all other habitats. These soils particularly acidified during the past period of high S deposition, and recovery from acidification might be more efficient than in spruce dominated forests. We note that climate warming can, in addition, increase base cation input to the soil via accelerated weathering and litter decomposition [34, 35], but significant effects of climate change on soil pH were only found in some of our study sites. Higher N deposition predominantly led to increasing soil C:N ratios until 2030 and 2050, but by 2100 most sites experienced negative effects. The effects were quite small (means < 1) and very likely irrelevant for plant species occurrence. A decrease in the soil C:N ratio in response to N deposition was found in numerous studies [21, 24]. However, N deposition may also stimulate plant growth, causing increases in soil C:N ratios [67]. Most of the study sites were N limited and exposed to relatively low N deposition, so that the implemented tree growth response to N deposition seems reasonable. As to plant species responses, we found what we expected: plant species preferring nutrient-rich sites increased to some extent (e.g. *Corylus avellana*, *Milium effusum*, *Senecio ovatus*, *Carex sylvatica*, *Athyrium filix-femina*, *Mercurialis perennis*). However, the resulting increase in the HSI means that additional N improved habitat suitability at these forest sites which might be due to the fact that N deficiency is still widespread in Austria, because of historic overuse and acidification during the last part of the 20^th^ century [64]. Notwithstanding the difficulty to explain the increase in HSI, deposition effects on species occurrence probabilities, and hence the HSI, were much smaller than effects from climate causing negative total changes in habitat suitability.

### Interactions of climate and N deposition

First, we note that the forest sites used for these analyses represent the major forest habitats in Austria with a wide distribution but do not cover many rarer habitats under conservation protection. Hence, only one of totally 8 of the EU directive Annex I priority habitats [5] could be included due to a lack of data. Our results suggests that while climate change will clearly lower the “favourable conservation status” of these Austrian forest habitats N deposition effects will be comparably weak. The reasons are twofold. First, N deposition in most of these forests will not exceed loads at which major changes in the soil chemistry occur, and, secondly, climate driven increase in N immobilisation in woody biomass will offset soil N enrichment, a result which is in line with Butler et al. [26]. Our results are also in line with observations showing that during the last decades European forests have become more nutrient deficient albeit N deposition was relatively high [68]. They showed that enhanced forest growth due to N deposition, climate warming and, possibly, CO_2_ fertilization increased the demand for N and other nutrients rendering soils more nutrient limited, underlining earlier hypothesis on progressive N limitation [69]. Indeed, incorporation of nutrient limitation other than N is a clear research field for the future. Observations are meagre but suggest that in some areas N (and S) deposition has caused changes in forest floor species composition [69, 70], while soil inventory data showed increases in pH but, together with needle leaf N concentrations, did not give an indication of large-scale soil N enrichment [64].

## Conclusions

Measures of habitat suitability, such as the HSI, have the advantage that they are directly relevant for habitat management and conservation policies [44, 71, 72]. According to the European Union conservation legislation, a protected habitat is considered to have “favourable conservation status” if “the specific structure and functions which are necessary for its long-term maintenance exist” [5]. The HSI is based on predictions from empirical niche functions of distinctive species in a habitat [41]. While plant species’ niche functions have been used extensively in climate impact assessments and seem to be well defined [71, 73], their usefulness in studying air pollution effects is just being discussed [74]. Here we applied niche functions implemented in the newly developed model PROPS, which uses mean annual T, annual total P, annual N deposition, soil C:N ratio, and the soil solution pH as predictor variables. We were able to model the soil chemistry in a reasonable way using the dynamic soil model VSD+ [40]. When comparing the results of PROPS with those from a second plant response model (BERN), which is based on an independent empirical data set and which is rather different as to its statistical approach [39], they showed a high correlation. Hence, the niche functions, which are implemented in the current version of PROPS are, in general, reliable and this is obvious, because they are statistical representations of observed species occurrence data. However, on a site scale, and particularly when management comes into play, predictions are inherently difficult and hard to validate, a fact that has caused considerable discussion in climate impact studies before [75] as has the applicability of empirical niche functions to perform future impact assessments [42] Nevertheless, we could show that climate and air pollution effects on habitat suitability significantly interact yet often in an idiosyncratic way. Owing to this complexity and the still high uncertainty in present knowledge, adaptation to protect forest habitat biodiversity, such as defined under the European Habitat Directive, will be challenging. Dynamic soil-plant models can play an important role as supporting tools to assess possible future trajectories.

## Supporting information

S1 Table List of all distinctive plant species for the study sites. (PDF)

S2 Table Methods used for soil and climate input data for VSD+. (PDF)

S3 Table Site- and scenario-specific effects of climate change and N deposition. (PDF)

S1 File VSD+ input data. (ZIP)

S2 File Methods and results for forest growth. (PDF)

S3 File Soil model validation. (PDF)

S4 File Future changes at the site level. (PDF)

S5 File Future changes in the occurrence probability of plant species. (PDF)
