# Supplementary material for: Climate and air pollution impacts on habitat suitability of Austrian forest ecosystems
Source: PLoS One. 2017 Sep 12;12(9):e0184194. doi: 10.1371/journal.pone.0184194 (PMC5595319; doi:10.1371/journal.pone.0184194)
Supplement: S1 Table — (PDF) [file pone.0184194.s001.pdf]

S1 Table. List of all distinctive plant species for the study sites

Site code	Distinctive species used in the calculation of HSI
IF_AT01	Ajuga reptans L., Anemone sylvestris L., Asarum europaeum L., Carex digitata L., Carex pilosa Scop., Carpinus betulus L., Cornus mas L., Corylus avellana L., Fagus sylvatica L., Galium odoratum (L.) Scop., Hedera helix L., Melica uniflora Retz., Poa nemoralis L., Prunus padus L., Pulmonaria officinalis L., Rhamnus catharticus L., Salvia glutinosa L., Viola reichenbachiana Jordan ex Boreau
IF_AT02	Campanula persicifolia L., Crataegus monogyna Jacq., Digitalis grandiflora Miller, Galium schultesii Vest, Genista tinctoria L., Lathyrus niger (L.), Bernh., Lathyrus vernus (L.) Bernh., Luzula luzuloides (Lam.) Dandy & Wilmo, Poa nemoralis L., Quercus petraea (Mattuschka) Liebl., Rosa canina L., Stellaria holostea L., Tanacetum corymbosum (L.) Schultz Bip., Veronica officinalis L., Vincetoxicum hirundinaria Medicus
IF_AT03	Abies alba Miller, Acer pseudoplatanus L., Ajuga reptans L., Athyrium filix-femina (L.) Roth, Bromus ramosus Hudson, Calamagrostis villosa (Chaix) J.F. Gme, Carex sylvatica Hudson, Dryopteris filix-mas (L.) Schott, Fagus sylvatica L., Festuca altissima All., Galium odoratum (L.) Scop., Geranium robertianum L., Gymnocarpium dryopteris (L.) Newman, Hieracium murorum L., Hordelymus europaeus (L.) C.O. Harz, Lamiastrum galeobdolon (L.) Ehrend. &,<br>Luzula sylvatica (Hudson) Gaudin, Milium effusum L., Mycelis muralis (L.) Dumort., Oxalis acetosella L., Phegopteris connectilis (Michx) Watt, Phyteuma spicatum L., Picea abies (L.) Karsten, Polygonatum verticillatum (L.) All., Prenanthes purpurea L., Senecio nemorensis ssp. fuchsii (C.C., Sorbus aucuparia L., Viola reichenbachiana Jordan ex Boreau
IF_AT04	Carex digitata L., Fagus sylvatica L., Galium odoratum (L.) Scop., Luzula pilosa (L.) Willd., Milium effusum L., Moehringia trinervia (L.) Clairv., Mycelis muralis (L.) Dumort., Oxalis acetosella L., Poa nemoralis L., Viola reichenbachiana Jordan ex Boreau
IF_AT05, IF_AT10	Abies alba Miller, Deschampsia flexuosa (L.) Trin., Fagus sylvatica L., Hieracium murorum L., Luzula luzuloides (Lam.) Dandy & Wilmo, Picea abies (L.) Karsten, Vaccinium myrtillus L.
IF_AT07	Berberis vulgaris L., Campanula persicifolia L., Carex alba Scop., Cephalanthera damasonium (Miller) Druc, Cephalanthera rubra (L.) L.C.M. Richar, Cyclamen purpurascens Miller, Fagus sylvatica L., Fraxinus excelsior L., Hieracium murorum L., Ligustrum vulgare L., Melittis melissophyllum L., Mercurialis perennis L., Pinus sylvestris L., Polygonatum odoratum (Miller) Druce, Primula vulgaris Hudson, Sorbus torminalis (L.) Crantz, Tanacetum corymbosum (L.) Schultz Bip., Viburnum lantana L., Vincetoxicum hirundinaria Medicus
IF_AT08	Abies alba Miller, Calamagrostis villosa (Chaix) J.F. Gme, Deschampsia flexuosa (L.) Trin., Dryopteris dilatata (Hoffm.) A. Gray, Fagus sylvatica L., Galeopsis bifida Boenn., Homogyne alpina (L.) Cass., Luzula sylvatica (Hudson) Gaudin, Oxalis acetosella L., Picea abies (L.) Karsten, Senecio nemorensis ssp. nemorensis, Sorbus aucuparia L., Stellaria nemorum L., Vaccinium myrtillus L., Veratrum album L.
IF_AT09	Acer pseudoplatanus L., Dryopteris dilatata (Hoffm.) A. Gray, Euphorbia amygdaloides L., Fagus sylvatica L., Festuca altissima All., Galium odoratum (L.) Scop., Gymnocarpium dryopteris (L.) Newman, Hordelymus europaeus (L.) C.O. Harz, Lamiastrum galeobdolon (L.) Ehrend., Melica uniflora Retz., Mercurialis perennis L., Mycelis muralis (L.) Dumort., Poa nemoralis L.
IF_AT11	Abies alba Miller, Acer pseudoplatanus L., Ajuga reptans L., Athyrium filix-femina (L.) Roth, Bromus ramosus Hudson, Calamagrostis villosa (Chaix) J.F. Gme, Carex sylvatica Hudson, Deschampsia flexuosa (L.) Trin., Dryopteris filix-mas (L.) Schott, Fagus sylvatica L., Festuca altissima All., Galium odoratum (L.) Scop., Geranium robertianum L., Gymnocarpium dryopteris (L.) Newman, Hieracium murorum L., Hordelymus europaeus (L.) C.O. Harz, Lamiastrum galeobdolon (L.) Ehrend., Luzula luzuloides (Lam.) Dandy & Wilmo, Luzula sylvatica (Hudson) Gaudin, Milium effusum L., Mycelis muralis (L.) Dumort., Oxalis acetosella L., Phegopteris connectilis (Michx) Watt, Phyteuma spicatum L., Picea abies (L.) Karsten, Polygonatum verticillatum (L.) All., Prenanthes purpurea L., Senecio nemorensis ssp. fuchsii (C.C., Sorbus aucuparia L., Vaccinium myrtillus L., Viola reichenbachiana Jordan ex Boreau
IF_AT12	Abies alba Miller, Betula pendula Roth, Betula pubescens ssp. carpatica (Willd, Blechnum spicant (L.) Roth, Calamagrostis villosa (Chaix) J.F. Gme, Cirsium palustre (L.) Scop., Deschampsia cespitosa (L.) Beauv., Deschampsia flexuosa (L.) Trin., Dryopteris carthusiana (Vill.) H.P. Fu, Dryopteris dilatata (Hoffm.) A. Gray, Juncus effusus L., Lysimachia vulgaris L., Picea abies (L.) Karsten, Sorbus aucuparia L., Trientalis europaea L., Vaccinium myrtillus L.
IF_AT13	Abies alba Miller, Acer pseudoplatanus L., Ajuga reptans L., Athyrium filix-femina (L.) Roth, Blechnum spicant (L.) Roth, Bromus ramosus Hudson, Calamagrostis villosa (Chaix) J.F. Gme, Carex sylvatica Hudson, Deschampsia flexuosa (L.) Trin., Dryopteris dilatata (Hoffm.) A. Gray, Dryopteris filix-mas (L.) Schott, Fagus sylvatica L., Festuca altissima All., Galium odoratum (L.) Scop., Geranium robertianum L., Gymnocarpium dryopteris (L.) Newman, Hieracium murorum L., Hordelymus europaeus (L.) C.O. Harz, Lamiastrum galeobdolon (L.) Ehrend., Luzula luzuloides (Lam.) Dandy & Wilmo, Luzula sylvatica (Hudson) Gaudin, Milium effusum L., Mycelis muralis (L.) Dumort., Oxalis acetosella L., Phegopteris connectilis (Michx) Watt, Phyteuma spicatum L., Picea abies (L.) Karsten, Polygonatum verticillatum (L.) All., Prenanthes purpurea L., Senecio nemorensis ssp. fuchsii (C.C., Sorbus aucuparia L., Vaccinium myrtillus L., Viola reichenbachiana Jordan ex Boreau
IF_AT14	Abies alba Miller, Athyrium filix-femina (L.) Roth, Carex sylvatica Hudson, Dryopteris carthusiana (Vill.) H.P. Fu, Galium rotundifolium L., Luzula pilosa (L.) Willd., Lysimachia nemorum L., Mycelis muralis (L.) Dumort., Oxalis acetosella L., Picea abies (L.) Karsten, Polygonatum verticillatum (L.) All., Prenanthes purpurea L., Senecio nemorensis ssp. fuchsii (C.C., Sorbus aucuparia L., Viola reichenbachiana Jordan ex Boreau
IF_AT15	Berberis vulgaris L., Calamagrostis varia (Schrader) Host, Carex alba Scop., Cephalanthera longifolia (L.) Fritsch, Cephalanthera rubra (L.) L.C.M. Richar, Cyclamen purpurascens Miller, Daphne mezereum L., Euphorbia amygdaloides L., Fagus sylvatica L., Fraxinus excelsior L., Helleborus niger L., Hepatica nobilis Schreber, Melittis melissophyllum L., Mercurialis perennis L., Mycelis muralis (L.) Dumort., Picea abies (L.) Karsten, Prenanthes purpurea L., Primula vulgaris Hudson, Salvia glutinosa L., Viburnum lantana L., Vincetoxicum hirundinaria Medicus
IF_AT16	Blechnum spicant (L.) Roth, Hieracium murorum L., Homogyne alpina (L.) Cass., Larix decidua Miller, Linnaea borealis L., Lonicera caerulea L., Luzula sieberi Tausch, Lycopodium annotinum L., Melampyrum sylvaticum L., Oxalis acetosella L., Picea abies (L.) Karsten, Pinus cembra L., Rhododendron ferrugineum L., Solidago virgaurea L., Vaccinium myrtillus L., Vaccinium vitis-idaea L.
IF_AT18	Abies alba Miller, Calamagrostis varia (Schrader) Host, Carex alba Scop., Cyclamen purpurascens Miller, Daphne mezereum L., Euphorbia amygdaloides L., Fragaria vesca L., Helleborus niger L., Hieracium murorum L., Mercurialis perennis L., Orthilia secunda (L.) House, Picea abies (L.) Karsten, Sesleria caerulea (L.) Ard.
IM_AT01	Abies alba Miller, Acer pseudoplatanus L., Aposeris foetida (L.) Less., Athyrium filix-femina (L.) Roth, Blechnum spicant (L.) Roth, Cardamine trifolia L., Carex alba Scop., Fagus sylvatica L., Galium odoratum (L.) Scop., Helleborus niger L., Huperzia selago (L.) Bernh. ex Schrank, Lamiastrum galeobdolon (L.) Ehrend., Luzula luzulina (Vill.) Dalla Torre, Lycopodium annotinum L., Oxalis acetosella L., Picea abies (L.) Karsten, Polygonatum verticillatum (L.) All., Prenanthes purpurea L., Vaccinium myrtillus L., Viola reichenbachiana Jordan ex Boreau
IM_AT02	Abies alba Miller, Acer pseudoplatanus L., Aposeris foetida (L.) Less., Campanula rotundifolia L., Cardamine enneaphyllos (L.) Crantz, Cardamine heptaphylla (Vill.) O.E. Sch, Carex alba Scop., Carex digitata L., Cyclamen purpurascens Miller, Dryopteris filix-mas (L.) Schott, Euphorbia amygdaloides L., Fagus sylvatica L., Festuca altissima All., Galium odoratum (L.) Scop., Helleborus niger L., Lonicera alpigena L., Lonicera nigra L., Mercurialis perennis L., Oxalis acetosella L., Picea abies (L.) Karsten, Polygala chamaebuxus L., Polystichum setiferum (Forsk+l) Woynar, Prenanthes purpurea L., Rosa pendulina L., Rubus idaeus L., Sesleria albicans Kit. ex Schultes, Valeriana montana L.
Sources:	Willner, W. (2002): Syntaxonomische Revision der südmitteleuropäischen Buchenwälder. Phytocoenologia 32: 337-453; Willner, W., Grabherr, G. (Hrsg.) (2007): Die Wälder und Gebüsche Österreichs – Ein Bestimmungswerk mit Tabellen. Elsevier Akademischer Verlag, Textband: 302 S. + Tabellenband: 297 p.; Knollová, I., Chytrý, M (2004): Oak-hornbeam forests of the Czech Republic: geographical and ecological approaches to vegetation classification. Preslia 76:291-311; Rolecek (2005): Vegetation types of dry-mesic oak forests in Slovakia. Preslia 77:241-261; Hartmann, F. K. und Jahn, G. (1967): Waldgesellschaften des mitteleuropäischen Gebirgsraumes nördlich der Alpen, Jena, 636 p.; Tüxen, R. (1937): Die Pflanzengesellschaften Nordwestdeutschlands. Mitt. flor.-soz. Arb.gem. 3:1-170. Hannover; Oberdorfer, E. (Hrsg.) (1992): Süddeutsche Vegetationsgesellschaften. Teil IV in 2 Bänden, Gustav-Fischer-Verlag, Jena Stuttgart New York
