# Supplementary material for: Climate and air pollution impacts on habitat suitability of Austrian forest ecosystems
Source: PLoS One. 2017 Sep 12;12(9):e0184194. doi: 10.1371/journal.pone.0184194 (PMC5595319; doi:10.1371/journal.pone.0184194)
Supplement: S2 Table — (PDF) [file pone.0184194.s002.pdf]

S2 Table. Overview of methods used in the soil model VSD+ (see also http://wge-cce.org/Methods_Models/Available_Models)

| parameter description | VSD+ parameter name | unit | details |
|---|---|---|---|
| Thickness of the soil compartment | thick | [m] | Mineral soil with < 80% coarse fraction; each horizon was reduced by its coarse fraction content |
| Average bulk density of the soil | bulkdens | [g.cm$^{-3}$] | weighted by horizon thickness; each horizon was reduced by its coarse fraction content |
| Soil water content | Theta | [m$^{3}$.m$^{-3}$] | Calculated with MetHyd (version 1.5.1) based on bulk density, soil texture, organic content and monthly temperature, precipitation and radiation |
| $CO_2$ pressure in the soil solution | pCO2fac | | according to CLRTAP (2004) [log10p$CO_2$ = -2.38 + 0.031 * Temp (°C)] |
| Ca in the parent material of a calcareous soil | parentCa | | taken from measurements in the lowest soil horizon |
| Clay content of the soil | Clay_ct | [%] | measured values |
| Average potential cation exchange capacity of the soil | CEC | [meq.kg$^{-1}$] | recalculated to a standard of pH = 6.5 according to CLRTAP (2004) |
| Cation exchange model | Excmod | - | Gaines-Thomas |
| log10 of selectivity constant for Al-BC exchange | lgKAlBC | - | calibrated within VSD+ |
| log10 of selectivity constant for H-BC exchange | lgKHBC | - | calibrated within VSD+ |
| Exponent in Al | expAl | - | default value 3 (gibbsite equilibrium) |
| log10 of Al equilibrium constant | lgKAlox | - | according to CLRTAP (2004) [lgKALox = 9.8602 – 1.6755 * log(OM) if 1.25 < OM < 100, lgKALox = 9.7 if OM <= 1.25 where OM is the organic matter in %] |
| Initial amount of C per unit area | Cpool_0 | [g.m$^{-2}$] | calibrated within VSD+ |
| Initial C:N ratio | CNrat_0 | - | calibrated within VSD+ |
| Organic acid dissociation model option | RCOOmod | - | mono-protic organic acid model |
| Concentration of organic acids (cOrgacids) | cRCOO | [mol.m$^{-3}$] | according to CLRTAP (2004) [cRCOO=m*DOC (mol.m$^{-3}$) where m=0.029 and DOC was estimated] |
| Parameters for organic acid dissociation | RCOOpars | - | estimated according to CLRTAP (2004) from pH-values |
| Average (soil) temperature | TempC | [C°] | Calculated with MetHyd (version 1.5.1) based on bulk density, soil texture, organic content and monthly temperature, precipitation and radiation |
| Percolation (precipitation surplus) | percol | [m.yr$^{-1}$] | Calculated with MetHyd (version 1.5.1) based on bulk density, soil texture, organic content and monthly temperature, precipitation and radiation |
| Weathering rate | Ca_we, Mg_we, K_we, Na_we | [eq.m$^{-3}$.yr$^{-1}$] | calibrated within VSD+ |
| Reduction factor of mineralisation rates due to moisture and temperature for the RothC C/N model | rf_miR | - | Calculated with MetHyd (version 1.5.1) based on bulk density, soil texture, organic content and monthly temperature, precipitation and radiation |
| Reduction factor of nitrification rates due to moisture and temperature | rf_nit | - | |
| Reduction of denitrification rates due to moisture and temperature | rf_denit | - | |
| Quality index of litterfall | QIlf | - | default value for forests is 0.25 |
| measured exchangeable base cations | EBC_obs | [%] | measured values averaged over the soil profil |
| measured soil C pool | Cpool_obs | [g.m$^{-2}$] | measured values summed over the soil profil |
| measured soil C:N ratio | CNrat_obs | - | measured values averaged over the soil profil |
| measured soil solution pH-value | pH_obs | - | either mean annual soil water measurements or from samples (soil soluted in water) |
| measured soil solution $SO_4^{2-}$ concentration | SO4_obs | [eq.m$^{-3}$] | mean annual soil water measurements |
| measured soil solution $NO_3^{-}$ concentration | NO3_obs | [eq.m$^{-3}$] | mean annual soil water measurements |
