# Supplementary material for: Climate and air pollution impacts on habitat suitability of Austrian forest ecosystems
Source: PLoS One. 2017 Sep 12;12(9):e0184194. doi: 10.1371/journal.pone.0184194 (PMC5595319; doi:10.1371/journal.pone.0184194)
Supplement: S3 Table — (PDF) [file pone.0184194.s003.pdf]

S3 Table. Effects on soil C:N ratio, pH-value, and Habitat Suitability Indicator (HIS) in climate change and N deposition scenarios. The ANOVA coefficients describe the difference between the mean values of all baseline climate model runs and the respective climate change scenario and the difference between the mean values of all Maximum Feasible Reduction deposition scenarios (MFR) and the respective N deposition scenario at each site. Positive coefficients represent increasing, negative coefficients decreasing effects. Time periods: 2030 (2025-35), 2050 (2045-55), 2100 (2090-2100); CLE: Current Legislation N deposition Scenario, B10: No change in N deposition after 2010.

		ANOVA coefficients C:N ratio						ANOVA coefficients soil pH						ANOVA coefficients HSI					
site	time	ECHAM A1B	ECHAM A2	ECHAM B1	HadCM3 A1B	CLE	B10	ECHAM A1B	ECHAM A2	ECHAM B1	HadCM3 A1B	CLE	B10	ECHAM A1B	ECHAM A2	ECHAM B1	HadCM3 A1B	CLE	B10
	2030	-0.44	-0.96	-0.66	-0.41	0.02	0.05	0.43	0.39	0.51	0.22	-0.01	-0.04	0	0.01	0	-0.03	0.02	0.05
IF_AT01	2050	-0.66	-0.82	-1.44	-0.6	0.02	0.03	0.18	0.27	0.49	0.17	-0.02	-0.09	-0.05	-0.04	-0.02	-0.05	0.03	0.05
	2100	-0.57	-0.37	-0.77	-0.61	-0.07	-0.16	0.11	0.01	0.1	0.01	-0.03	-0.15	-0.08	-0.08	-0.06	-0.08	0.02	0.03
	2030	-0.62	-0.73	-1.06	-0.2	0.04	0.11	0	-0.01	0	0.01	0	-0.01	-0.01	0	0	-0.01	0.01	0.01
IF_AT02	2050	-0.99	-1.25	-1.24	-1.05	0.06	0.11	0	-0.01	-0.02	0	0	-0.02	-0.03	-0.02	-0.02	-0.03	0.01	0.02
	2100	-0.48	-0.8	-0.88	-0.72	-0.09	-0.25	0.01	0.02	0.01	0.03	0	-0.02	-0.06	-0.06	-0.04	-0.06	0.01	0.02
	2030	0.12	0.15	0.15	0.59	0.03	0.08	0	-0.04	0.05	-0.01	0	-0.06	0	-0.01	0	-0.04	0	0.01
IF_AT03	2050	0.67	0.63	0.32	1.11	0.05	0.1	-0.02	-0.05	0.05	-0.04	0	-0.06	-0.07	-0.05	-0.03	-0.09	0	0
	2100	2.34	3.51	1.47	3.76	0	-0.01	-0.17	-0.36	-0.05	-0.18	0.01	-0.09	-0.15	-0.17	-0.12	-0.19	-0.01	-0.01
	2030	0.11	0.2	0.14	1.18	0.02	0.03	0	0	0	0	0	-0.02	0	-0.01	0	-0.01	0.02	0.03
IF_AT04	2050	0.9	0.65	0.76	2.81	0.04	0.05	0	-0.01	0.01	0	0	-0.02	0	0	0	0	0.02	0.02
	2100	6.2	6.54	3.35	8.59	0.01	0.01	-0.02	-0.02	-0.01	-0.02	0	-0.03	0	0	0	0.01	0.02	0.02
	2030	-0.08	0.03	0.13	0.9	0.02	0.05	0.25	0.31	-0.07	0.05	-0.02	-0.09	0	0.01	0	0	0.02	0.04
IF_AT05	2050	0.48	0.65	0.51	1.31	0	-0.01	-0.04	-0.06	0.05	-0.02	-0.02	-0.07	0.01	0.01	0	0.01	0.01	0.03
	2100	2.51	4.05	1.93	4.52	-0.13	-0.28	0.17	0.48	0.45	0.59	-0.02	-0.12	0.01	-0.02	0.02	0	0.04	0.07
	2030	-0.36	0.06	0.44	1.13	0.03	0.07	0	0	0	0.01	0	-0.01	0.01	0	-0.01	-0.03	0	-0.01
IF_AT07	2050	0.5	0.79	0.27	2.41	-0.04	-0.22	0.02	0.02	0	0.01	0	-0.02	-0.01	-0.02	0	-0.04	0.01	0.02
	2100	2.27	2.91	1.83	2.92	-0.41	-1.36	0.03	0.06	0.03	0.04	0	-0.02	-0.04	-0.04	-0.03	-0.04	0.01	0.02

S3 Table continued

		ANOVA coefficients C:N ratio						ANOVA coefficients soil pH						ANOVA coefficients HSI					
site	time	ECHAM A1B	ECHAM A2	ECHAM B1	HadCM3 A1B	CLE	B10	ECHAM A1B	ECHAM A2	ECHAM B1	HadCM3 A1B	CLE	B10	ECHAM A1B	ECHAM A2	ECHAM B1	HadCM3 A1B	CLE	B10
IF_AT08	2030	-0.02	-0.28	-0.05	0.18	0.03	0.06	0.02	-0.02	-0.01	-0.09	-0.02	-0.11	0	0	0	-0.03	0	0.01
	2050	0.34	-0.08	-0.05	0.27	0.01	-0.01	-0.1	-0.06	-0.02	-0.04	-0.05	-0.19	-0.04	-0.03	-0.01	-0.03	0	0
	2100	0.87	0.87	0.41	1.37	-0.17	-0.38	-0.08	-0.13	-0.08	-0.13	-0.06	-0.29	-0.07	-0.08	-0.05	-0.08	-0.01	-0.01
IF_AT09	2030	0.07	-0.11	0.07	0.33	0.02	0.08	-0.01	0	-0.01	0.02	0	0	0	0	0	0	0	0
	2050	0.48	0.17	0.32	0.81	0.03	0.1	-0.01	0	-0.01	0.03	0	-0.01	0.01	0	0	0.01	0	-0.01
	2100	1.04	1.08	0.7	1.29	0.01	0.01	0.03	0.08	-0.01	0.09	0	-0.01	0.01	0.02	0.01	0.02	-0.01	-0.01
IF_AT10	2030	-0.09	0.04	0	0.59	0.06	0.09	-0.02	0.01	-0.02	-0.02	-0.01	-0.03	0	-0.02	0	-0.07	0.01	0.02
	2050	0.44	0.29	0.22	0.71	0.06	0.08	0.09	-0.06	0	-0.08	-0.01	-0.03	-0.1	-0.06	-0.01	-0.06	0	0.01
	2100	1.41	2.07	0.92	2.29	-0.13	-0.21	0.03	0.3	0.11	0.16	-0.03	-0.08	-0.04	-0.08	-0.07	-0.07	0	0
IF_AT11	2030	0.01	0.02	0.07	0.54	0.08	0.18	-0.03	0.02	0.04	0.16	-0.02	-0.12	-0.01	-0.01	-0.02	-0.07	0	0.01
	2050	0.76	0.34	0.48	1.25	0.1	0.16	0.1	0.06	0.02	-0.03	-0.02	-0.12	-0.06	-0.04	-0.03	-0.05	0	0.01
	2100	2.67	3.26	1.55	3.73	-0.21	-0.47	0.03	0.22	0.15	0.18	-0.02	-0.11	-0.06	-0.07	-0.02	-0.08	0.02	0.02
IF_AT12	2030	0.12	0.05	-0.12	0.84	0.01	0.01	0	0	-0.04	0	0	-0.01	0	0	0	0.02	0	0
	2050	1.46	0.69	0.55	1.87	0.01	0.01	0.13	-0.02	0	-0.02	0	-0.01	0.03	0.01	0.01	0.02	0	0
	2100	4.99	5.56	2.2	6.56	-0.1	-0.1	0.26	0.36	0.25	0.37	0	-0.03	0.01	0.01	0.04	0.01	0	0
IF_AT13	2030	-0.04	0.03	0.1	-0.01	0.02	0.06	0.02	0.04	0.07	0.05	-0.01	-0.09	0	0	0	0	0	0
	2050	0.23	0.26	0.22	0.47	0.03	0.09	0.05	0.02	0.08	0.09	-0.01	-0.07	0	0	0	0	0	0
	2100	0.51	0.77	0.39	0.68	0.03	0.07	0.13	0.06	0.06	0.14	-0.02	-0.11	0	0	0	0	0	0
IF_AT14	2030	0.05	0.43	0.43	0.94	0.03	0.06	0	-0.04	-0.01	-0.03	-0.01	-0.05	0	0	0	0.01	0	0
	2050	0.82	0.7	0.89	1.7	0.06	0.11	-0.02	-0.03	0	0	-0.01	-0.05	0.03	0.02	0.01	0.01	0	0
	2100	3.98	4.35	3.13	5.02	0.07	0.11	-0.14	-0.19	-0.1	-0.13	-0.02	-0.09	-0.11	-0.11	-0.1	-0.12	0.01	0.02
IF_AT15	2030	0.22	0.23	0.28	0.88	0.02	0.05	0	0	0	0.01	0	0	0	0	0	-0.01	-0.01	-0.01
	2050	1.39	0.57	0.9	1.78	0.03	0.05	0.02	0.01	0	0.01	0	0	-0.01	0	-0.01	-0.01	0.01	0
	2100	5.07	6.07	3.08	6.23	-0.08	-0.2	0.03	0.03	0.02	0.03	0	0	0	-0.01	0.01	0	0.01	0.01

S3 Table continued

site	time	ANOVA coefficients C:N ratio						ANOVA coefficients soil pH						ANOVA coefficients HSI					
		ECHAM A1B	ECHAM A2	ECHAM B1	HadCM3 A1B	CLE	B10	ECHAM A1B	ECHAM A2	ECHAM B1	HadCM3 A1B	CLE	B10	ECHAM A1B	ECHAM A2	ECHAM B1	HadCM3 A1B	CLE	B10
IF_AT16	2030	0.16	0.1	0.54	0.84	0.01	0	0	0	0	0	0	-0.01	0	0	0	0	0	0
	2050	1.47	0.53	1.03	2.17	0.01	0.01	0	0	0	0	0	-0.01	0	0	0	0	0	0
	2100	4.84	5.17	3.18	5.97	-0.02	-0.01	-0.01	-0.02	-0.01	-0.01	0	-0.01	0	0	-0.01	0	0.01	0
IF_AT18	2030	0.22	0	0.25	0.58	0.01	0.03	0	0	0	0.01	0	0	-0.01	0.01	0	-0.01	0.03	0.09
	2050	0.51	0.34	0.45	1.33	-0.01	-0.05	0.02	0.01	0.01	0.02	0	0	0	0	0	-0.02	0.03	0.09
	2100	3.83	4.75	2.02	5.56	-0.2	-0.44	0.03	0.03	0.02	0.03	0	0	-0.03	-0.04	-0.01	-0.04	0.02	0.05
IM_AT01	2030	0.2	0.06	0.15	0.78	0.14	0.29	0	0	0	0.01	0	0	0.01	0	0.01	0.02	0	0
	2050	0.88	0.29	0.56	1.11	0.28	0.46	0.02	0.01	0	0.01	0	0	0.02	0	0.02	0.03	0	0
	2100	1.99	2.41	1.21	2.46	0.17	0.26	0.03	0.03	0.02	0.03	0	0	0.05	0.04	0.03	0.05	0	0
IM_AT02	2030	0.16	0	0.1	0.61	0.11	0.19	0	0	0	0.01	0	0	0	0	0	-0.02	-0.01	-0.02
	2050	0.65	0.18	0.39	0.72	0.14	0.21	0.02	0.01	0	0.01	0	0	-0.01	-0.01	0.01	0	-0.01	-0.02
	2100	1.23	1.48	0.75	1.49	-0.05	-0.1	0.03	0.03	0.02	0.03	0	0	0.03	0.02	0.01	0.04	-0.01	-0.01
