# Supplementary material for: Climate and air pollution impacts on habitat suitability of Austrian forest ecosystems
Source: PLoS One. 2017 Sep 12;12(9):e0184194. doi: 10.1371/journal.pone.0184194 (PMC5595319; doi:10.1371/journal.pone.0184194)
Supplement: S2 File — (PDF) [file pone.0184194.s005.pdf]

S2. Methods and results for forest growth

Methods

Since both climate change and N deposition are supposed to change tree growth and thereby the uptake of N by trees and input via litterfall, a scaling of these input variables was done comparable to De Vries and Posch (2011). N and base cation uptake as well as C and N in litterfall were scaled according to a reference situation, i.e. mean values between 1970 and 1990, when the core of the forest yield tables were obtained, by

$$A_i = A_{i,ref} \cdot f_{temp} \cdot f_{cue} \cdot f_{drought} \cdot f_{N_{dep}}$$

where, f_{temp}, f_{cue}, $f_{drought}$, and f_{Ndep} are reduction or enhancement factors, A_i is the amount of C, N or base cations in litterfall, or N uptake by trees, and $A_{i,ref}$ refers to the period 1970–1990. The direct effect of temperature, f_{temp}, was calculated according to equation 2 in de Vries and Posch (2011) and ranged between 1.004 and 1.112 in the different climate scenarios, meaning that the expected temperature rise can stimulate growth by up to 11% as compared to 1970–1990.

The temperature effect on carbon use efficiency (CUE) was modelled according to Piao et al. (2010):

$$f_{cue} = \frac{\left(1 - \frac{R_a}{GPP}\right)_T}{\left(1 - \frac{R_a}{GPP}\right)_{T_{ref}}} \text{ with } \frac{R_a}{GPP} = 0.0012 \cdot T^2 - 0.0263 \cdot T + 0.664$$

where R_a is the autotrophic respiration and GPP is the gross primary production. f_{cue} is >1 at moderate temperature increases of 2-3°C and <1 above those changes. Mean f_{cue} values ranged between 1 and 1.012 under the different climate scenarios (see S3). Because of large uncertainties in the magnitude of the effects of CO_2, nutrient limitation apart from N, and ozone on tree growth, we did not account for them.

The effect of drought stress on tree growth was modelled as:

$$f_{drought} = \frac{AET}{AET_{ref}}$$

where AET (actual evapotranspiration) is the current and AET_{ref} the reference evapotranspiration (1970–1990), i.e. such that a decrease in evapotranspiration decreases growth (De Vries and Posch 2011). AET is computed with the MetHyd model (Bonten et al. 2016). Since our sites are, on average moist sites, where AET is close to potential evapotranspiration, mean $f_{drought}$ is mostly positive, ranging between 0.996 and 1.102.

Similar to temperature increase, N deposition is enhancing or decreasing tree growth (via f_{Ndep}). A roughly 1-2% growth stimulation with every 1 kg N $ha^{-1} yr^{-1}$ has been observed in European forests (Laubhann et al. 2009, Solberg et al. 2009). Since we only modelled sites with N deposition < 30 kg N $ha^{-1} yr^{-1}$, decreasing effects of excessive N deposition, as in (De Vries and Posch 2011), were not considered. N deposition has decreased since the reference period 1970–1990 and decreases even further in our two emission reduction scenarios. Since N-stimulated growth very likely lags

behind changes in deposition, we use a conservative 1% growth reduction with every kg N ha^{-1} yr^{-1} reduction after the year 2010.

Results

The scenario means by 2100 of the product of all *f*-factors, f_{all}, describing the reduction or enhancement of tree growth compared to the period 1970–1990, ranged between 1.18 and 1.39, i.e. tree growth increased by 18% to 39% compared to the 1970–1090 period (Table 1 in the manuscript). Although variation between sites was considerable, only in one site (IF_AT02) was f_{all}<1 in 2100. This low f_{all} was a result of very low $f_{drought}$ factors. Among the different growth factors, f_{temp} was the most important, followed by $f_{drought}$, f_{Ndep}, and f_{cue}.

References

Bonten, L. T. C., G. J. Reinds, and M. Posch. 2016. A model to calculate effects of atmospheric deposition on soil acidification, eutrophication and carbon sequestration. Environmental Modelling & Software **79**:75-84.

De Vries, W. and M. Posch. 2011. Modelling the impact of nitrogen deposition, climate change and nutrient limitations on tree carbon sequestration in Europe for the period 1900–2050. Environmental Pollution **159**:2289-2299.

Laubhann, D., H. Sterba, G. J. Reinds, and W. De Vries. 2009. The impact of atmospheric deposition and climate on forest growth in European monitoring plots: An individual tree growth model. Forest Ecology and Management **258**:1751-1761.

Piao, S., S. Luyssaert, P. Ciais, I. A. Janssens, A. Chen, C. Cao, J. Fang, P. Friedlingstein, Y. Luo, and S. Wang. 2010. Forest annual carbon cost: a global-scale analysis of autotrophic respiration. Ecology **91**:652-661.

Solberg, S., M. Dobbertin, G. J. Reinds, H. Lange, K. Andreassen, P. G. Fernandez, A. Hildingsson, and W. de Vries. 2009. Analyses of the impact of changes in atmospheric deposition and climate on forest growth in European monitoring plots: A stand growth approach. Forest Ecology and Management **258**:1735-1750.
