# Supplementary material for: Climate and air pollution impacts on habitat suitability of Austrian forest ecosystems
Source: PLoS One. 2017 Sep 12;12(9):e0184194. doi: 10.1371/journal.pone.0184194 (PMC5595319; doi:10.1371/journal.pone.0184194)
Supplement: S3 File — (PDF) [file pone.0184194.s006.pdf]

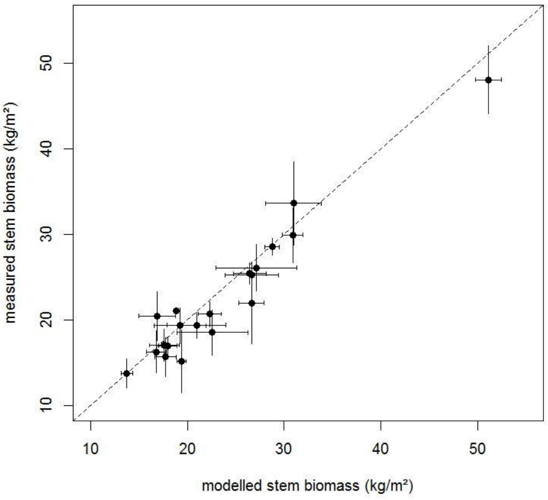

S3 Figure A. Comparison between measured and modelled stem biomass (mean and standard error; years 1995 to 2009). Sites IF_AT11 and IM_AT02 are not included because of either significant harvest or sparse biomass data.

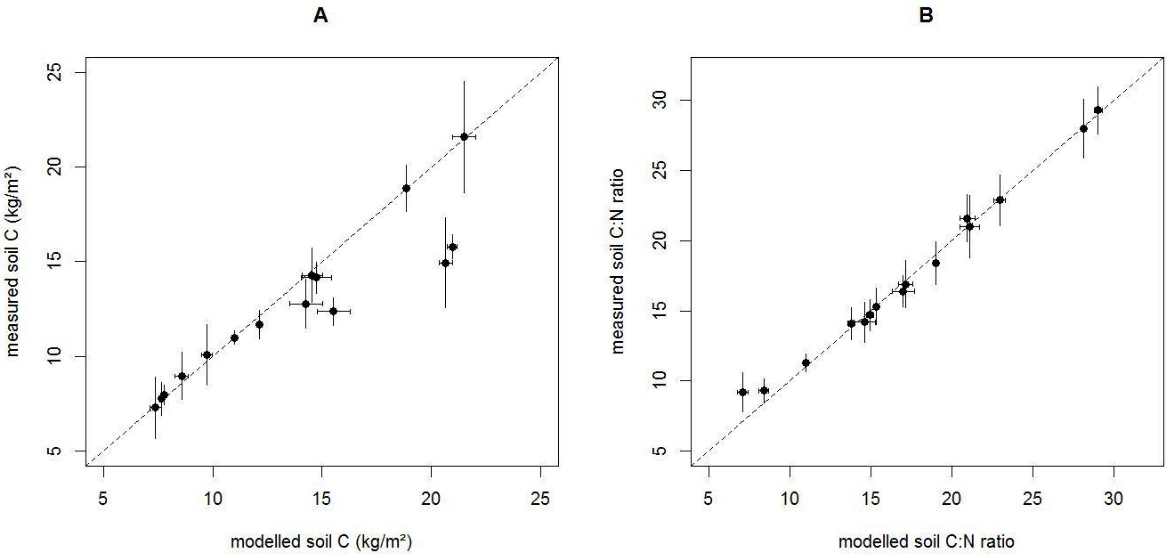

S3 Figure B. Comparison between measured and modelled soil C pool (A) and soil C:N ratios (B) (mean and standard error). Sites IF_AT08, IF_AT11, and IF_AT16 were excluded because of significant harvest before soil inventory. Mean and standard errors of measured values were calculated from replicated soil profiles in 2008 or 2005. Five year means and standard deviations were calculated from the modelled results for comparison.

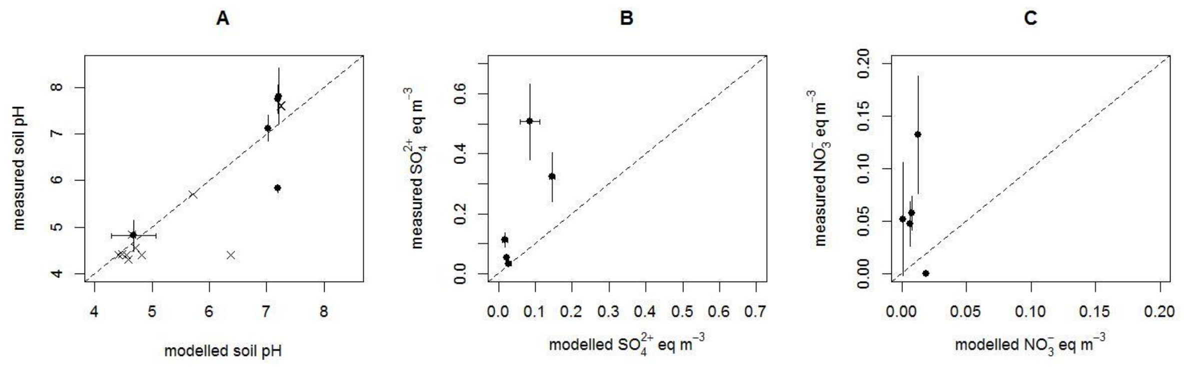

S3 Figure C. Comparison of measured and modelled soil solution pH (A), [SO_4^{2-}] (B), and [NO_3^-] (C) (mean and standard error). Only sites IF_AT02, IF_AT09, IF_AT15, IM_AT01 and IM_AT02 are shown for which measurements for more than 3 years exist. A: in addition to soil water pH from lysimeter samples, results for soil samples are shown with a cross symbol.
