# Supplementary material for: Climate and air pollution impacts on habitat suitability of Austrian forest ecosystems
Source: PLoS One. 2017 Sep 12;12(9):e0184194. doi: 10.1371/journal.pone.0184194 (PMC5595319; doi:10.1371/journal.pone.0184194)
Supplement: S4 File — (PDF) [file pone.0184194.s007.pdf]

S4 Table A. Model results for combined climate (2xA1B, A2, and B1) and deposition (B10, CLE, MFR) scenarios. Mean values ± standard errors of the mean of temperature (T), precipitation (P), nitrogen deposition (N_{dep}), sulphur deposition (S_{dep}), soil solution pH, soil C:N ratio, and the Habitat Suitability Index (HSI) for four time slices (2010: 2005-15; 2030: 2025-35; 2050: 2045-55; 2100: 2090-2100). Significant ANOVA p-values (<0.05) are in bold.

	T (°C)	P (mm yr^{-1})	N_{dep} (kg N ha^{-1} yr^{-1})	S_{dep} (kg N ha^{-1} yr^{-1})	soil pH	soil C:N	HSI
2010	8.5 ±0.09	1046 ±18.7	10.1 ±0.4	2.8 ±0.06	5.7 ±0.08	19.7 ±0.39	0.2 ±0.006
2030	8.8 ±0.1	1042 ±17.4	8.2 ±0.37	1.7 ±0.06	5.7 ±0.07	20.1 ±0.41	0.18 ±0.006
2050	9.5 ±0.1	1071 ±18.9	8 ±0.36	1.6 ±0.06	5.7 ±0.07	21 ±0.45	0.17 ±0.005
2100	11.2 ±0.13	970 ±19	8 ±0.36	1.6 ±0.06	5.9 ±0.07	23.1 ±0.56	0.14 ±0.005
time	**<0.001**	**0.001**	**<0.001**	**<0.001**	0.209	**<0.001**	**<0.001**
time:clim	**<0.001**	0.109	0.999	0.999	0.999	0.958	0.96
time:depo	0.999	0.999	**<0.001**	**<0.001**	0.999	0.999	0.97
time:clim:depo	0.999	0.999	0.999	0.999	0.999	0.999	0.999

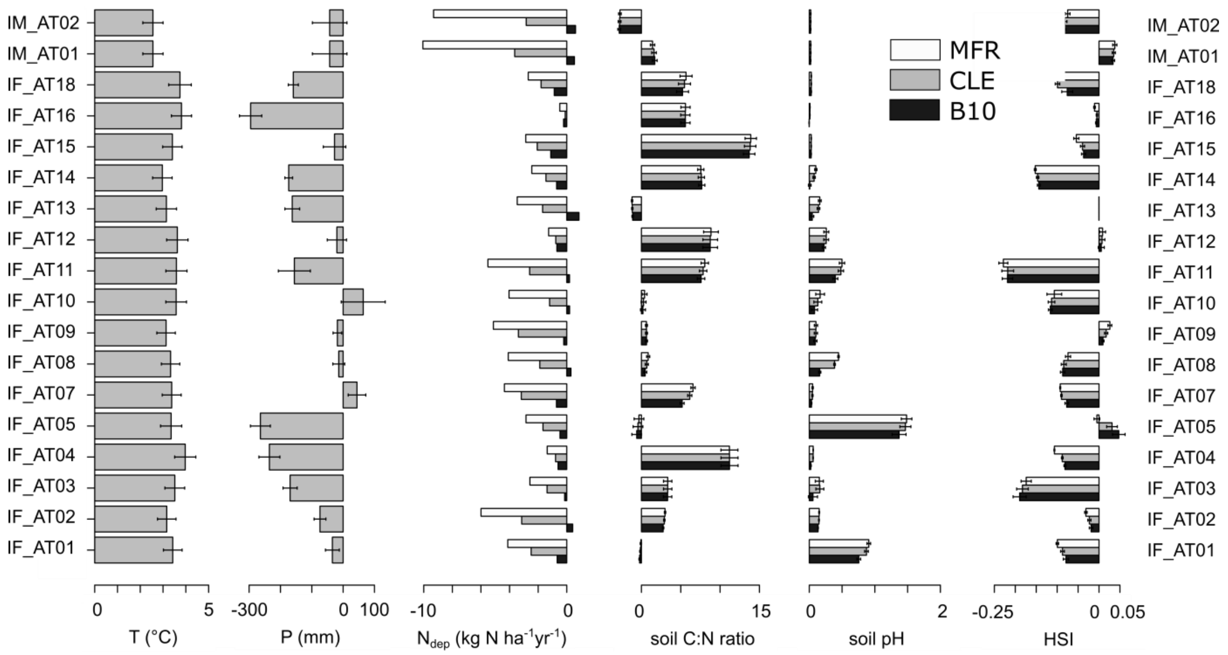

S4 Figure A. Relative changes between 2010 and 2100 in annual mean temperature (T), precipitation (P), N deposition (N_{dep}), soil solution pH (pH), soil C:N ratio, and HSI. Bars show mean values of all climate scenarios, error bars are standard errors of mean values.
