# Supplementary material for: Climate and air pollution impacts on habitat suitability of Austrian forest ecosystems
Source: PLoS One. 2017 Sep 12;12(9):e0184194. doi: 10.1371/journal.pone.0184194 (PMC5595319; doi:10.1371/journal.pone.0184194)
Supplement: S5 File — (PDF) [file pone.0184194.s008.pdf]

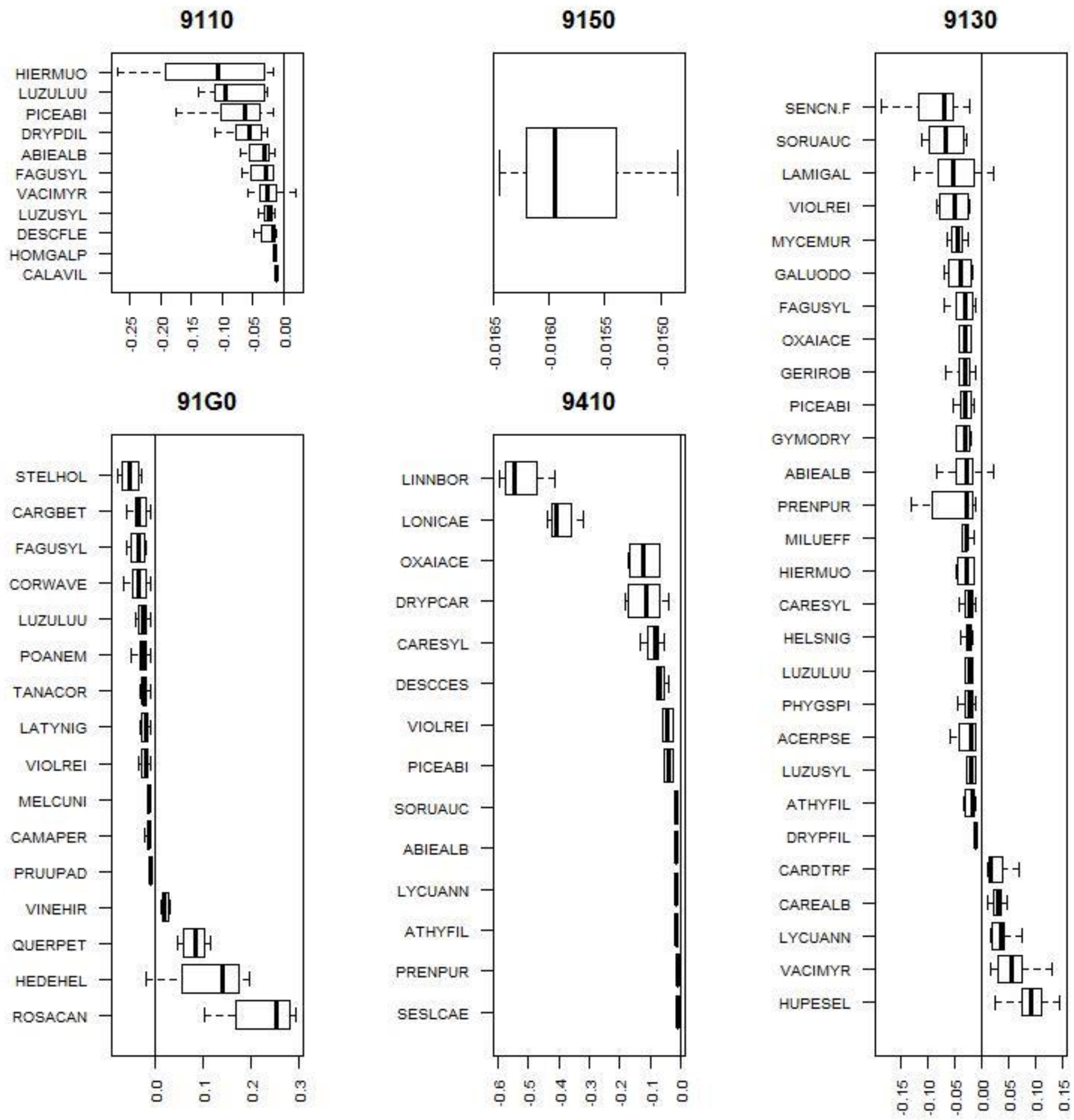

S5 Figure A. Effects in species occurrence probabilities between climate scenarios (A1B, A2, B1) and the baseline climate for the year 2100 in 5 forest habitat types. A positive value indicates a higher probability under the climate scenario. Each climate scenario was run with three N deposition scenarios (B10, CLE, MFR) at each site. 9110: Luzulo-Fagetum beech forests, 9150: Medio-European limestone beech forests of the Cephalanthero-Fagion, 9130: Asperulo-Fagetum beech forests, 91G0: Pannonic woods with *Quercus petraea* and *Carpinus betulus*, 9410: Acidophilous *Picea* forests of the montane to alpine levels (Vaccinio-Piceetea). Only species with effects > 1% are shown. Full species names and lists of character species per site see S1 Table.

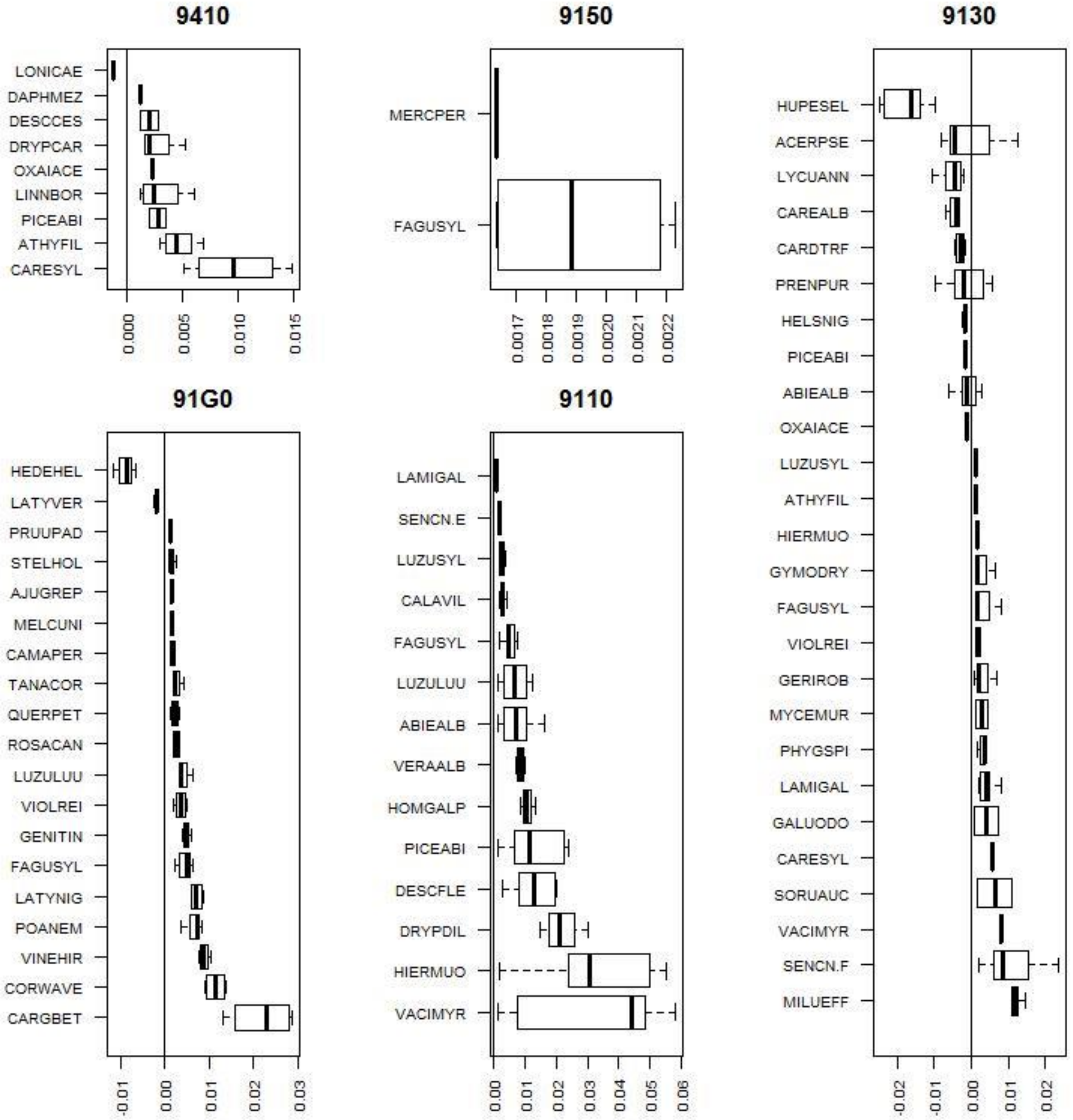

S5 Figure B. Effects in species occurrence probabilities between deposition scenarios (CLE, B10) and the deposition scenario with the strongest reduction (MFR) for the year 2100 in 5 forest habitat types. A positive value indicates a higher probability in the CLE and B10 scenario. Each deposition scenario was run with four climate scenarios (ECHAM5-A1B, HADCM3-A1B, ECHAM5-A1, ECHAM5-B2) at each site. 9110: Luzulo-Fagetum beech forests, 9150: Medio-European limestone beech forests of the Cephalanthero-Fagion, 9130: Asperulo-Fagetum beech forests, 91G0: Pannonic woods with *Quercus petraea* and *Carpinus betulus*, 9410: Acidophilous *Picea* forests of the montane to alpine levels (Vaccinio-Piceetea). Only species with effects > 1% are shown. Full species names and lists of character species per site see S1 Table.
